# Supplementary material for: Inflammatory factors and risk of lung adenocarcinoma: a Mendelian randomization study mediated by blood metabolites
Source: Front Endocrinol (Lausanne). 2024 Aug 27;15:1446863. doi: 10.3389/fendo.2024.1446863 (PMC11384989; doi:10.3389/fendo.2024.1446863)
Supplement: Supplementary file 1 [file DataSheet1.zip › supplementary files/S2/GCST90200604/forest.pdf]

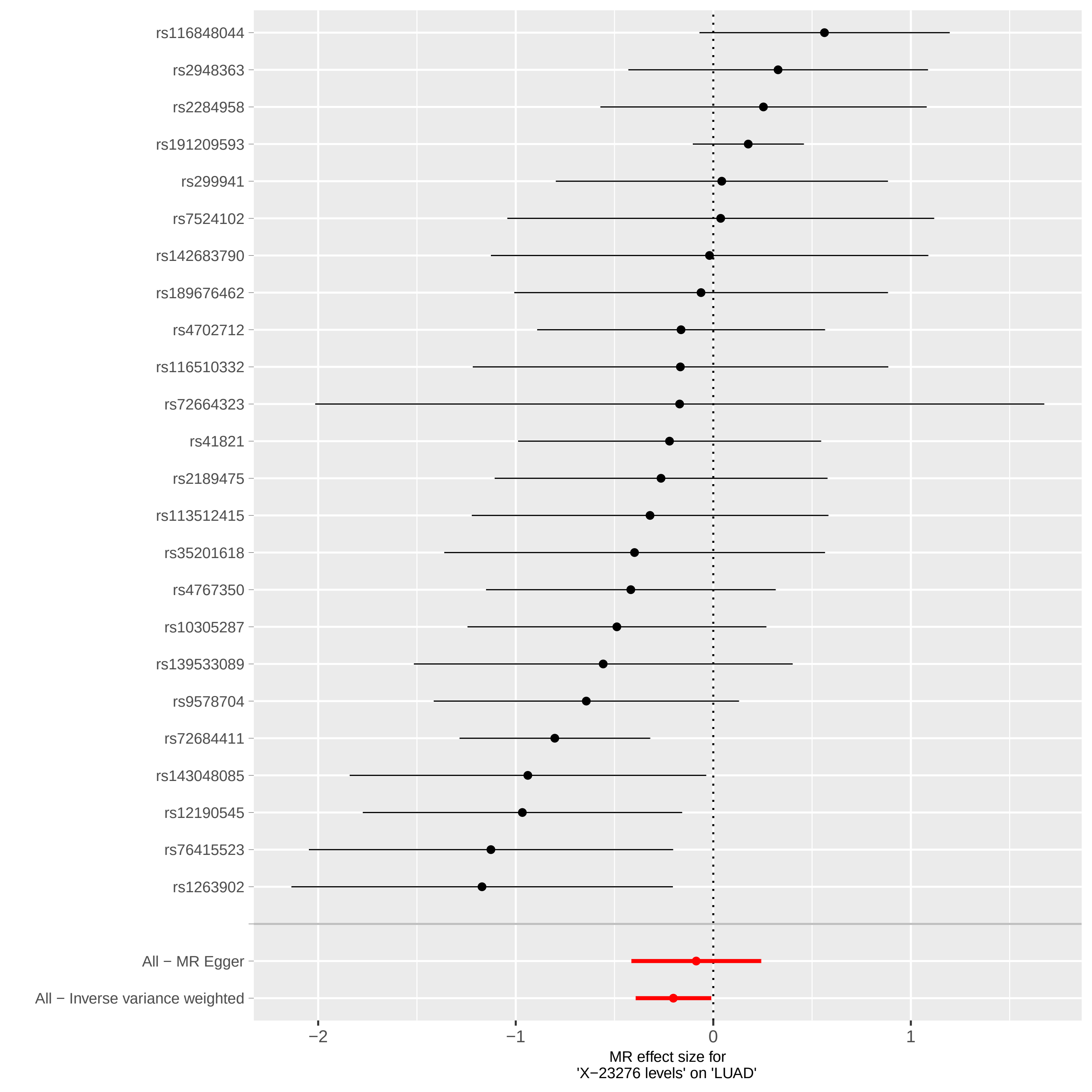

rs116848044
rs2948363
rs2284958
rs191209593
rs299941
rs7524102
rs142683790
rs189676462
rs4702712
rs116510332
rs72664323
rs41821
rs2189475
rs113512415
rs35201618
rs4767350
rs10305287
rs139533089
rs9578704
rs72684411
rs143048085
rs12190545
rs76415523
rs1263902
All − MR Egger
All − Inverse variance weighted
−2
−1
0
1
MR effect size for
'X−23276 levels' on 'LUAD'
